# Modeling of switching mechanism in GeSbTe chalcogenide superlattices

**DOI:** 10.1038/srep12612

**Published:** 2015-07-29

**Authors:** Xiaoming Yu, John Robertson

**Affiliations:** 1 Engineering Dept, University of Cambridge, Cambridge CB2 1PZ, UK

## Abstract

We study the switching process in chalcogenide superlattice (CSL) phase-change memory materials by describing the motion of an atomic layer between the low and high resistance states. Two models have been proposed by different groups based on high-resolution electron microscope images. Model 1 proposes a transition from Ferro to Inverted Petrov state. Model 2 proposes a switch between Petrov and Inverted Petrov states. For each case, we note that the main transition is actually a vertical displacement of a Ge layer through a Te layer, followed by a lateral motion of GeTe sublayer to the final, low energy structure. Through calculating energy barriers, the rate-determining step is the displacive transition.

Phase change materials based on Ge, Sb and Te (GST) are promising candidates for next generation non-volatile random-access memories. They have the merits of scalability, endurance, good retention and fast switching speed while changing between the memory states^1^,^2^. Recently, a new type of phase change memory device called ‘interfacial phase change memory’ (iPCM) or ‘chalcogenide superlattice’ (CSL) was proposed^3^,^4^,^5^. In this case, the phase transition is between two crystalline structures, rather than between an amorphous and a crystalline phase. The CSLs consist of hexagonal (GeTe)_n_(Sb_2_Te_3_)_m_ layer units deposited along a growth direction. The idea is to constrain the switching transition to motion in one dimension instead of three dimensions, so that the transition may consume less energy than the traditional process involving an amorphous to crystal transition^5^.

There have been a number of experimental demonstrations of the CSL memory^5^,^6^,^7^,^8^,^9^,^10^,^11^, but the switching mechanism is so far not fully defined at an atomic level. There have been several suggestions to explain how the atoms are manipulated, including charge injection^12^, electric field^6^,^13^, magnetic field^6^, thermal activation^7^ and polarization dependent optical control^14^. However, the atomic structures of the low resistance state (LRS) and high resistance states (HRS) are still not fully agreed by different groups, so the atomic mechanism needs further definition. At present, two models have been proposed, based on analysis of high-resolution electron microscope images and symmetry arguments. Referring to Fig. 1, Model 1 proposes a transition from a Ferro LRS to an Inverted-Petrov structure HRS^7^,^8^,^15^. Model 2 proposes a transition between a Petrov LRS and an Inverted-Petrov HRS^11^,^12^,^16^. As the electrical field is applied normal to the layers, both models have focused primarily on switching as a vertical movement of Ge atoms through a Te atom layer. However, considering the atomic structures of the more stable phases, it is clear that an additional lateral movement is also required. Thus, we calculate here the overall switching transitions at an atomic level and the energy barriers involved.

We show that the mechanism must be a two-steps process. In step 1, the external electric field acting in the vertical [001] direction causes a vertical displacement of Ge layers along this direction. However, this does not lead to one of the low energy structures. Thus, we note that the full transition must also include as step 2 a lateral motion of the GeTe sublayer to the final, lower energy structure. By evaluating the energy barrier^17^,^18^,^19^ of this two-step transition, we provide a new view on the atomic movement of the phase change between HRS and LRS.

The GeSbTe CSLs have the basic formulae (GeTe)_n_(Sb_2_Te_3_)_m_. The simplest CSL supercell consists of hexagonal (GeTe)_2_(Sb_2_Te_3_) units (n = 2, m = 1). This unit can adopt four different basic structures in which the primary bonds in different layers are aligned, according to the ordering of Ge, Sb and Te layers, and depending on the position of the van der Waals gap between Te-Te layers^6^, as shown in Fig. 1. In the Kooi structure, the van der Waals (vdW) gap is between the two Sb_2_Te_3_ blocks. In the Ferro structure, the (GeTe)_2_ block has a Ge-Te-Ge-Te sequence and the vdW gap is between the (GeTe)_2_ and Sb_2_Te_3_ block. In the Petrov structure, the (GeTe)_2_ block has Ge-Te-Te-Ge sequence and the vdW gap is between the Te layers in this (GeTe)_2_ block. In the Inverted-Petrov structure, the (GeTe)_2_ block has a Te-Ge-Ge-Te sequence and the vdW gaps are between the GeTe layers and the Sb_2_Te_3_ blocks.

The relative stability of these four structures depends on temperature, which we have calculated from the phonon dispersion spectrum and plotted the enthalpy diagram against temperature in Fig. 2. From this, we see that the Kooi structure has the lowest enthalpy at 0 K, as previously found by Tominaga *et al*.^6^. However, the Kooi phase is unfavorable for switching. We see that by raising the temperature by 200 K, the enthalpy of the Kooi state increases and the Ferro structure becomes the more stable phase. This was a motivation to deposit the CSLs at ~250 °C, to favor the Ferro phase^6^.

High resolution TEM images of various CSLs have been measured; some show a Ferro-like ordering^7^, whereas some show a Petrov like order^11^. Interestingly one work simultaneously shows both Ferro and Petrov-like ordering in the same image^11^.

From the above, a simple vertical movement of Ge planes from the Ferro state or Petrov state does not give the basic Inverted-Petrov state, but a variant of it (see Fig. 3). In order to study the intermediate states, we classify the related structures to the Ferro (F), Petrov (P) and Inverted Petrov (IP) structures but with different intra-layer orderings. Taking IP as an example, there are three different structures, IP_0 which is the original IP structure, plus two new variants, IP_1 and IP_2, as Fig. 3. Similarly from F_0 we can generate F_1, F_2 and F_3, and from P_0 we can generate P_1 and P_2.

The variant structures are fully relaxed at 0 °K and the total energies are calculated, as given in Table 1, together with the lattice constants of the primitive cells. The F_0 is set to 0 eV as a reference. According to our results, the four (original) structures with the aligned bonds have the lowest total energy compared to the new structures.

We now consider the full switching transition for both model 1 and model 2. The IP_0 structure is the HRS for both models and the LRS is either F_0 or P_0. We suppose that although the intermediate states can be reached through vertical atomic displacement, the final HRS and LRS structures are still from the most stable candidates. As the lateral motion does not change the stacking order of atoms, we conclude that this movement happens between two structures with same group name. The completed SET and RESET cycle for each model is summarized below, and in Fig. 4(a,b).

In model 1:
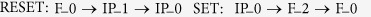


In model 2:
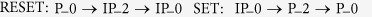


Note that lateral motion is needed for both SET and REST.

It should be noted that some works give confusing assignments. For example, the LRS or ‘SET’ structure in Bang^11^ is F_2 in our notation, while the TEM image is F_0. Some schematics are shown at an angle to suggest a relation to an umbrella-flip transition^20^. However, the so-called 4-fold Ge site in Ohyanagi^9^ actually breaks bonds under energy minimization to become the IP_1 state. F_0 is the only state that has primary and secondary Ge-Te bonds in sequence as in rhombohedral GeTe.

We then calculate the energy barriers for each process. As the vertical flip is a process where the Ge and Te layers cross each other, the highest energy point on the transition path is where the Ge and Te atoms are in the same layer. In this case, the distance between atoms is lowest and the energy barrier is high, between 2.56 eV and 3.10 eV, Table 2 and Fig. 4(c).

The lateral movement is more complicated than the vertical one. There are two cases for bulk movement. We note that the Ge and Te atoms exchange their positions with their nearest bonded atoms during the lateral motion. Thus, we focus on a single Ge-Te sublayer in the primitive cell. A typical sublayer viewed from [001] direction is plotted in Fig. 5(a). The primitive cell has been expanded to a 2 × 2 supercell along x and y directions. In the initial structure, the Ge atoms are on site A while Te atoms are on site B. After the movement, the Ge atoms move to site B and Te atoms are on site A.

From Fig. 5(a), the Ge atoms have three nearest Te sites and these three directions are equivalent by the hexagonal symmetry of the CSL. Once the Ge atoms begin to move, the Te atoms then have two choices: move towards where the Ge came from or move along the Ge atom stream to a nearby Ge vacancy (Fig. 5a). In the first case, an atom will roll over the top of an adjacent atom (‘over-head’), which will compress the surrounding Sb_2_Te_3_ and GeTe layers and only one bond is conserved in the movement, Fig. 5(b). In the second situation, the atoms move in plane, an atom breaks one bond to its neighbor, but conserves two other bonds, and moves ‘snake-like’ in the xy-plane. Once reaching the next low energy site, the atom rebonds with its new neighbor. As two bonds per atom are conserved in choice 2, this motion has a low energy barrier of only 0.44 eV, about 0.5 eV less than the 0.92 eV for the overhead rolling motion. The corresponding energy barriers are listed in Table 2. The overall energy barrier for the whole transition varies from 2.56 eV to 3.10 eV, which is close to the experiment value of 2.3 eV^10^. As the vertical flip motion has by far the larger energy barrier, this will dominate the switching process.

In model 1, only one GeTe sublayer is involved in the lateral movement. However, for model 2, both two GeTe sublayers must move to a lower energy state. We compared the energy barrier of the movement for a one at a time case with the situation where two GeTe bilayers move together. Interestingly, the energy barrier is similar. For example, in the SET process of model 2, the energy barrier for one by one movement is 0.51 eV, and the simultaneous movement ranges from 0.48 eV and 0.60 eV, which means this movement is not sequence-dependent.

We also calculated the situation where the lateral motion occurred by the migration of Ge vacancies. This is considered because Takaura^11^ noted that a Ge deficit in the CSLs favored the observation of switching. The Ge vacancy can move across a sublayer by flipping bonds^19^. The barrier for Ge vacancy diffusion is found to be 0.44 eV also. In Fig. 5(c,1) a Ge atom labeled blue next to a Ge vacancy moves as arrowed to a site where it overcoordinates with Te sites (Fig. 5c,2). The Ge atom also bonds to a Ge site in the sublayer below. The Ge atom then moves further down to the vacancy site, Fig. 5(c,3). Overall, the Ge and the Ge vacancy have exchanged places. Thus, the lateral motion could occur by a more concerted ‘snake-like’ motion of GeTe sublayers, or by a Ge vacancy migration. We have modeled the switching for the case of no atomic inter-mixing between the GeTe and Sb_2_Te_3_ sublattices. This stands as an end-point if there is inter-mixing.

Thus, we note that the switching mechanism in the all solid-state CSLs is different to that for the bulk switching between amorphous and liquid phases involving melting. There, the transition may occur by a local ‘umbrella flip’ of a Ge from a 6-fold to a 4-fold coordinated site^20^, or by an off-center displacement of a Ge from a 6-fold to a 4-fold site^21^.

In conclusion, the switching transition in CSL materials occurs as a 2-step process, a vertical flip of Ge sublayers through Te sublayers driven by the applied field, followed by a lower energy lateral motion of the GeTe sublayers back to the more stable configurations. The energy barrier for the vertical atomic flip of Ge layers is a high energy process with a barrier of 2.56 to 3.1 eV, and is followed by a lateral motion of GeTe sublayers with barriers of 0.44 eV, whether by vacancy diffusion, or by a more concerted bulk motion.

## Methods

### Ab initio simulations

We perform the simulations using the plane wave, density function theory (DFT) CASTEP code^22^ using ultrasoft plane-wave pseudopotentials. The plane wave represent the valence electrons Ge 4s^2^ 4p^2^, Sb 5s^2^ 5p^3^ and Te 5s^2^ 5p^4^. The exchange correlation functional uses the generalized gradient approximation (GGA) of Perdew-Burke-Ernzerhof (PBE)^23^. Spin-orbit coupling is not included. The van der Waals correction is added to the GGA using the Grimme scheme with the DFT-D2 correction function^24^ to give the correct inter-layer distances. The plane-wave cut-off energy is 400 eV. We use a 7 × 7 × 1 Monkhorst-Pack grid for the k-point mesh in the total energy calculation and geometry relaxation. The structures have been fully relaxed and the total energy is converged to under 1 × 10^−6^ eV per atom. The value for acceptable residual force is 0.03 eV Å^−1^ and the stress tolerance is 0.05 GPa. The thermodynamic properties such as enthalpy are calculated from the phonon dispersion simulation using density functional perturbation theory (DFPT)^25^,^26^. For the energy barrier calculation, we use the transition state search algorithm. The complete linear synchronous transitions (LST) and quadratic synchronous transitions (QST) simulation has been performed for finding the transition state^27^.

## Additional Information

**How to cite this article**: Yu, X. and Robertson, J. Modeling of switching mechanism in GeSbTe chalcogenide superlattices. *Sci. Rep*. **5**, 12612; doi: 10.1038/srep12612 (2015).

## Figures and Tables

**Figure 1:**
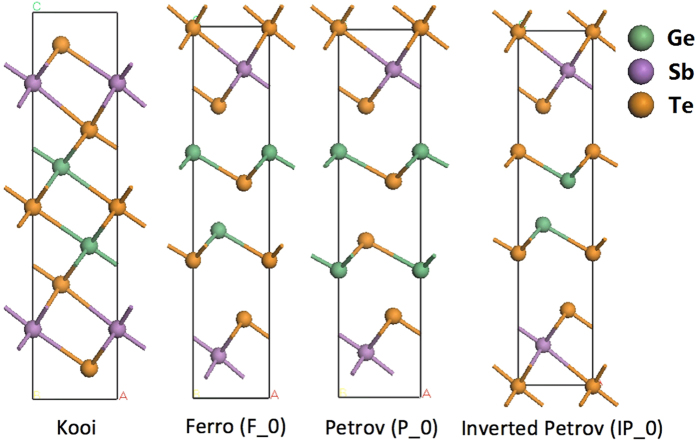
Four simplest CSL configurations based on (GeTe)_2_(Sb_2_Te_3_) layers. Note the alignment of primary bonding between layers.

**Figure 2:**
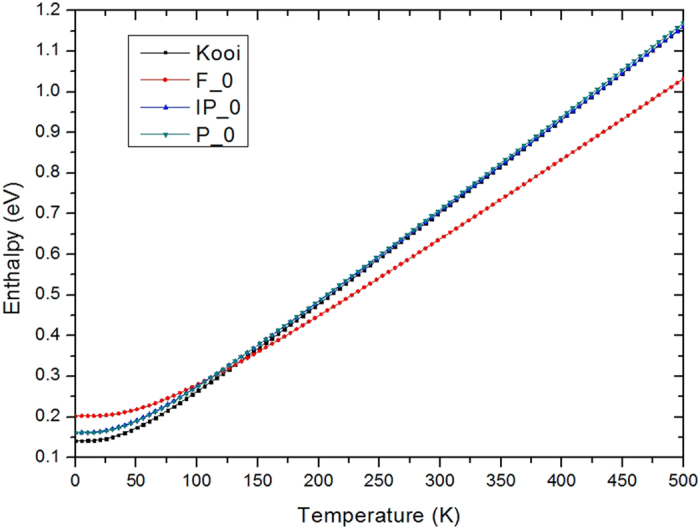
Variation of enthalpy against temperature. The Ferro phase has the highest enthalpy at low temperature, but it gets more stable above 125K.

**Figure 3:**
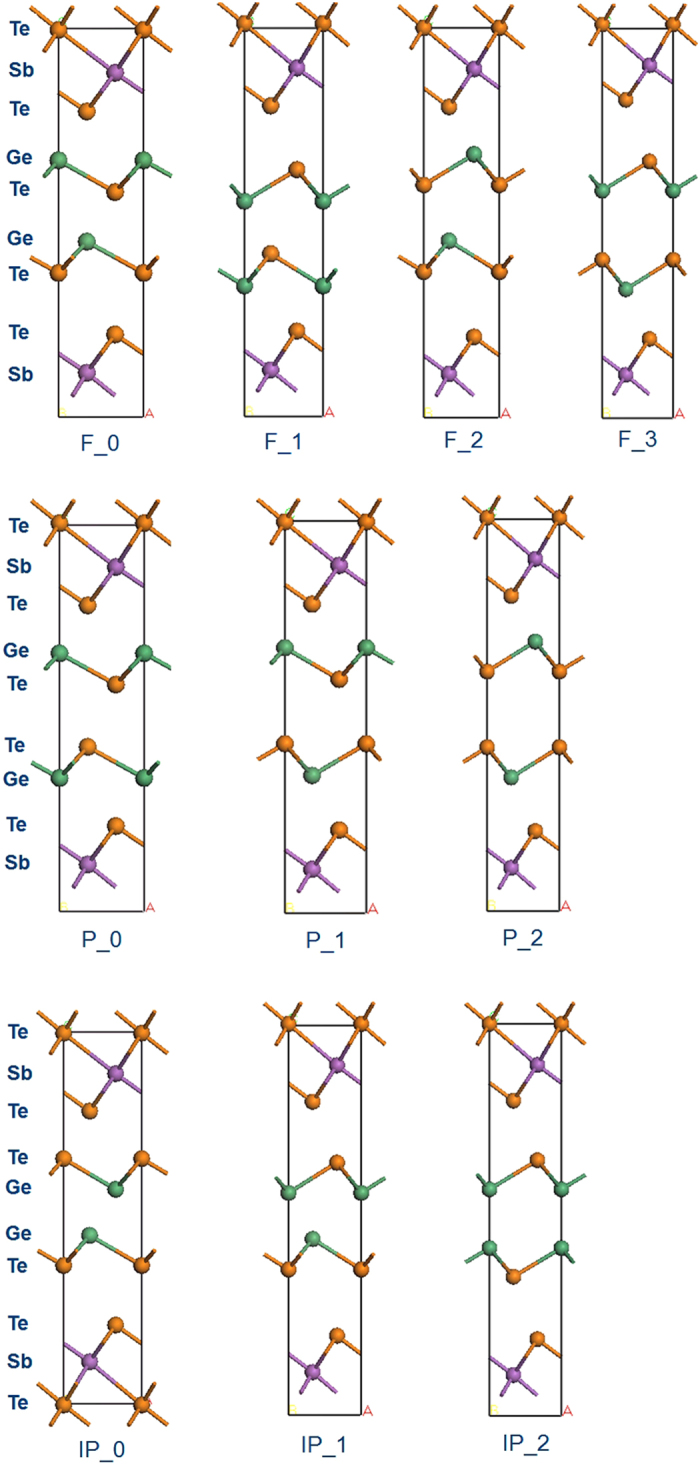
Top: Four different in-plane variants of the basic Ferro structure with Ge-Te-Ge-Te or Te-Ge-Te-Ge order. Middle: Three variants of the basic Petrov structure with Ge-Te-Te-Ge order. Bottom: Three variants of the basic Inverted Petrov structure with Te-Ge-Ge-Te order. Due to the symmetry and the constraint of the movement along z-axis, there are only three possibilities for Petrov and Inverted Petrov.

**Figure 4:**
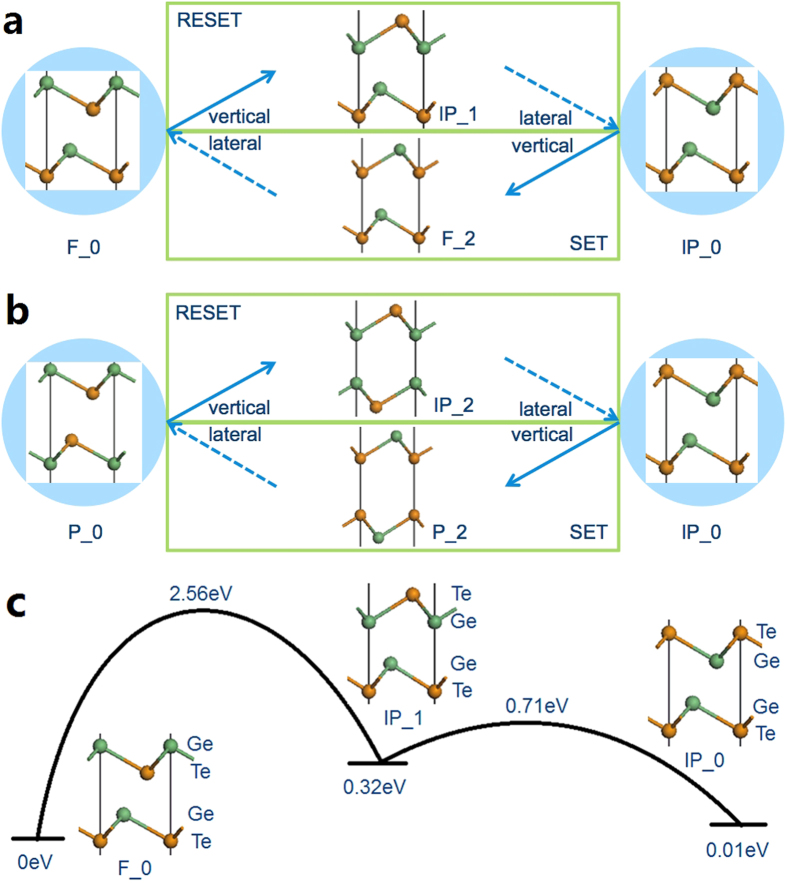
(**a**,**b**) The proposed routes of SET and RESET for model 1 and model 2. The solid arrow is the vertical flip under the external electric field, and the dash arrow is the following lateral atom diffusion. (**c**) Schematic of energy barriers for the transition between F_0 and IP_0. The energy values are referred to that of F_0 which is set to 0e V. Green atom: Ge, Orange atom: Te. The Sb_2_Te_3_ block is as in Fig. 1 and is left out here.

**Figure 5:**
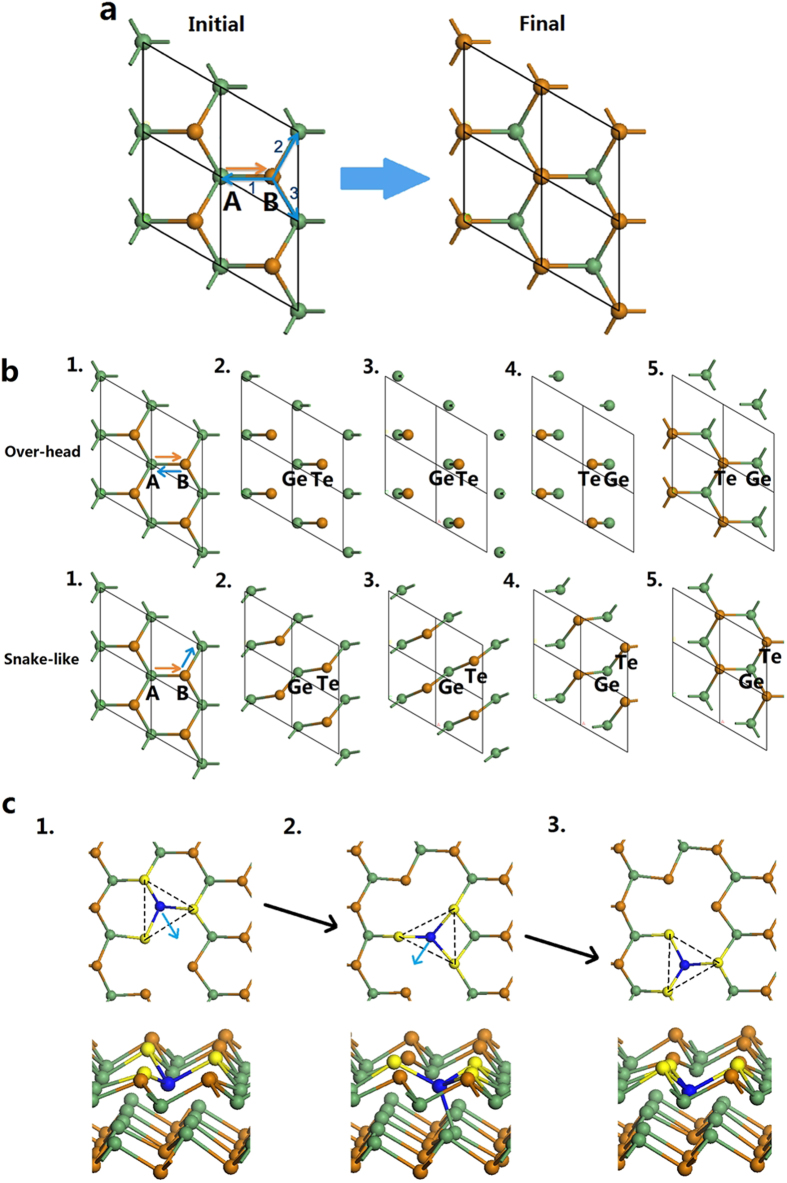
Three paths for lateral motion of GeTe layer. (**a**) Overall process. Ge atom can transfer from A site to B site without bias (orange arrow), while the Te atom has three routes to choose, 1, 2 and 3 (blue arrows). Due to the symmetry, route 2 and 3 are the same which is different with route 1. (**b**) Atomic pathways for the over-head lateral motion when Te follow route 1 and for snake-like movement when choosing route 2 or 3 are shown between the initial and final structures through three internediate snapshots. (**c**) Schematic diffusion path of a Ge vacancy. A Ge adjacent to the vacancy (brown) moves across to an overcoordinated site, and then down into the vacancy site. The moving Ge becomes 4-fold coordinated at the intermediate state.

**Table 1:** Lattice constants of the various CSL structures given in Fig. 3, and their total energies referred to the F_0 state of Fig. 1, as calculated by the van der Waals corrected PBE density functional.

Structure	a (a = b) (Å)	c (Å)	Total energy referred to F_0 (eV)
F_0	4.20	17.25	0.00
F_1	4.16	17.84	0.10
F_2	4.14	18.47	0.30
F_3	4.13	19.59	0.58
IP_0	4.14	18.13	0.01
IP_1	4.11	19.03	0.32
IP_2	4.13	19.53	0.75
P_0	4.18	17.31	0.07
P_1	4.15	17.90	0.17
P_2	4.10	19.36	0.49
Kooi	4.20	17.07	−0.10

**Table 2:** Calculated energy barriers within the van der Waals corrected PBE functional, for the vertical and lateral atomic motions in the RESET and SET transitions, in model 1 and model 2, as defined in Fig. 4(a,b).

Mode l	Memory process	Motion type	Reactant and product	Energy barrier (eV)
Model 1	RESET	Vertical flip	F_0− > IP_1	2.56
		Lateral motion	IP_1− > IP_0	0.39
	SET	Vertical flip	IP_0− > F_2	2.84
		Lateral diffusion	F_2− > F_0	0.62
Model 2	RESET	Vertical flip	P_0− > IP_2	2.59
		Lateral diffusion	IP_2− > IP_0	0.05
	SET	Vertical flip	IP_0− > P_2	3.10
		Lateral diffusion	P_2− > P_0	0.51
